# Association between Preoperative Exercise Tolerance, Comorbidities, and Survival Rates in Patients with Pancreatic Cancer

**DOI:** 10.31662/jmaj.2025-0105

**Published:** 2025-06-20

**Authors:** Makoto Onji, Shingo Kozono, Asuka Nakai, Shinji Kakizoe, Koichi Naito

**Affiliations:** 1 Department of Rehabilitation, Kitakyushu Municipal Medical Center, Kitakyushu, Japan; 2 Department of Surgery, Kitakyushu Municipal Medical Center, Kitakyushu, Japan; 3 Department of Physical Therapy, Faculty of Medical Science, Nagoya Aoi University, Aichi, Japan

**Keywords:** pancreatic cancer, exercise tolerance, comorbidity, survival rate

## Abstract

**Introduction:**

Despite treatment advancements, pancreatic cancer continues to have the lowest 5-year survival rate and a high age-adjusted mortality. Limited physical and functional reserves often restrict therapeutic options. Although the 6-minute walk distance (6MWD) and the Charlson Comorbidity Index (CCI) are established prognostic markers, their combined prognostic utility remains unexplored. This study evaluated the prognostic value of a composite index (6MWD-CCI) in patients who underwent pancreatic resection.

**Methods:**

This retrospective study included 85 patients with pancreatic cancer who underwent resection between July 2019 and September 2022. Preoperative 6MWD (<400 m) and CCI scores were used to classify patients into three 6MWD-CCI risk groups (low, middle, and high). Physical, nutritional, and frailty parameters were also assessed. Kaplan-Meier and Cox regression analyses were performed to evaluate survival outcomes, adjusting for confounders.

**Results:**

During a median follow-up of 802 days, 27 patients (31.8%) died. Low 6MWD, high CCI, and elevated 6MWD-CCI risk levels were significantly associated with reduced survival. The composite 6MWD-CCI demonstrated strong prognostic value, outperforming individual metrics even after adjustment for confounders.

**Conclusions:**

The 6MWD-CCI is a practical and predictive tool for pancreatic cancer prognosis, integrating physical function and comorbidity burden to enhance risk stratification. Its incorporation into clinical workflows could improve preoperative planning. Validation through larger studies is recommended.

## Introduction

The 5-year relative survival rate for pancreatic cancer ranges from approximately 4.3% to 10% in Japan and other countries ^(1), (2)^, markedly lower than that for other highly invasive cancers, such as esophageal cancer (41.5%) and liver cancer (35.8%). Although age-adjusted mortality rates for most malignancies are declining, pancreatic cancer uniquely exhibits the highest rate of increase among both male and female populations ^(3)^. This malignancy carries an exceptionally poor prognosis, with high recurrence rates persisting even after curative resection, underscoring the urgent need for preventive therapeutic interventions. Although neoadjuvant and adjuvant chemotherapy are recommended therapeutic strategies, their application is often limited by various factors, particularly reduced performance status (PS) ^(4)^. Maintaining PS for consistent treatment implementation requires sufficient individual functional reserve capacity, including physical function. Metrics assessing physical status, such as cardiopulmonary function, exercise capacity, and frailty in patients with cancer, have demonstrated significant associations with survival rates ^(5), (6), (7)^. Regarding preoperative exercise tolerance, the six-minute walk distance (6MWD), assessed via the six-minute walk test (6MWT), has served as a prognostic marker in several cancers, including esophageal cancer ^(8), (9)^, hepato-pancreato-biliary cancer ^(6)^, and lung cancer ^(10)^.

The Charlson Comorbidity Index (CCI) is a validated tool for assessing comorbidity, with higher scores demonstrating a significant correlation with increased mortality ^(11)^. The CCI remains a widely utilized clinical instrument due to its incremental validity in clinical measurements and superior inter-rater reliability ^(12)^. Studies exploring the relationship between CCI and long-term outcomes have revealed that elderly patients with elevated CCI scores experience higher 90-day mortality rates following pancreaticoduodenectomy ^(13)^. Although numerous studies have independently examined the relationship between long-term outcomes and either 6MWD or CCI, no prior investigations have evaluated survival outcomes using a combined assessment of exercise tolerance and CCI. Thus, this study aimed to examine the associations between survival outcomes and three key parameters in patients with pancreatic cancer: preoperative 6MWD, CCI, and a novel composite index integrating both measures (6MWD-CCI).

## Materials and Methods

### Study design and participants

This retrospective study analyzed 133 patients who underwent pancreatic resection at our institution between July 2019 and September 2022. The final analysis included 85 patients after excluding those diagnosed with non-pancreatic cancer (n = 39) and individuals with stage 0 (n = 2), stage III (n = 1), stage IV (n = 3), and total pancreatectomy (n = 3) due to their significant impact on survival rates and surgical outcomes.

Opt-out consent was implemented through our hospital’s website in accordance with the Helsinki Declaration. The publicly available study outline allowed potential participants to decline research participation. This study was approved by the ethics committee of our hospital (approval number. 202401006).

### Exercise tolerance

Exercise tolerance was assessed using the 6MWT, which measured the 6MWD. The test was performed on a 30-meter walkway, strictly adhering to the American Thoracic Society (ATS) guidelines ^(14)^. Before testing, participants received a comprehensive explanation of the test’s purpose and procedures. Instructions emphasized walking the maximum possible distance within 6 min, with the option to voluntarily terminate the test if fatigue or other adverse symptoms occurred. Following the ATS protocol, standardized verbal encouragement was provided at regular intervals, and participants received time updates every minute. To ensure patient safety, continuous monitoring of oxygen saturation and heart rate was performed using a pulse oximeter. Based on prior research, a cut-off value of 400 m was adopted for the analysis ^(6)^.

### Comorbidities

The CCI was used to assess comorbidities. Developed in 1987, the CCI remains one of the most widely utilized tools for assessing comorbid conditions and is considered the gold standard for evaluating comorbidity burden in clinical research ^(11)^. The CCI assigns specific points to various diseases to quantify comorbidity severity, including diabetes mellitus, hypertension, pneumonia, chronic obstructive pulmonary disease, hepatitis B, hepatitis C, acute myocardial infarction, coronary artery disease, cerebrovascular disease, heart valve dysfunction, sepsis, chronic kidney disease, heart failure, aortic aneurysm, peripheral vascular disease, peptic ulcer disease, dementia, chronic pulmonary disease, connective tissue disease, mild liver disease, hemiplegia, moderate or severe renal disease, and moderate or severe liver disease Completely resolved conditions (e.g., a history of pneumonia) and inactive surgical histories (e.g., a history of cholecystectomy) were not included as comorbidities. Based on previous studies, comorbidity scores were categorized as follows: low (score = 0), middle (score = 1-2), and high (score ≥3) ^(13)^. Although the CCI includes a category for “solid tumor;” this was excluded from scoring, as pancreatic cancer was the primary focus, consistent with prior research ^(6)^.

### Combination of exercise tolerance and comorbidities (6MWD-CCI)

A new composite index, the 6MWD-CCI, was defined using established cut-off values: low risk (6MWD ≥ 400 m and CCI categorized as low), middle risk (6MWD < 400 m or CCI categorized as middle or high), and high risk (6MWD < 400 m and CCI categorized as middle or high).

### Measurement items

A handheld dynamometer (μ-tas, Anima Co., Ltd.) measured knee extension strength with participants seated and hip and knee joints flexed at 90°. The highest value from two measurements of each leg was recorded. A digital grip strength meter (grip D, Takei Scientific Instruments) measured handgrip strength in the standing position with the elbows extended. The maximum value from two measurements of each hand was adopted. Body weight normalized all muscle strength measurements to account for physique differences ^(15)^. These physical function tests, including the 6MWD, were conducted in all patients a few days before surgery. Basic information comprised age, sex, body mass index, Brinkman index, nutritional status using the Geriatric Nutrition Risk Index (GNRI), and frailty evaluated by the Clinical Frailty Scale (CFS). The GNRI served as an indicator of nutritional status and provided a simple and accurate screening tool incorporating weight, height, and serum albumin levels ^(16)^. Frailty was defined as a CFS score of ≥4 ^(17)^. Medical data included neoadjuvant chemotherapy (NAC) status, clinical stage, and hospital stay length. Surgical data encompassed procedure type, operative time, total infusion volume, blood transfusion requirements, and postoperative complications (Clavien-Dindo grade Ⅲ or higher) ^(6)^. All data were retrospectively reviewed.

### Statistical analyses

The primary outcome of this study was overall survival at the study’s end date. The survival time for each patient was calculated from the date of surgery to the date of death from any cause or the end of the follow-up period. To preliminarily exclude potential confounding between 6MWD and CCI, we examined their correlation and subsequently compared CCI’s three categories (low, middle, and high) with 6MWD. Kaplan-Meier curves were created for exercise tolerance, comorbidities, and the 6MWD-CCI, followed by log-rank tests to assess survival rate differences. Cox proportional hazards analysis was conducted to investigate associations with survival rates, with independent variables including 6MWD, CCI, and 6MWD-CCI. Adjustments were made for known factors such as age, clinical stage, surgical procedure, postoperative complications (model 1), and frailty (model 2). To avoid issues with sample size and multicollinearity, postoperative complications and frailty were separately analyzed. Furthermore, to verify the predictive accuracy of 6MWD-CCI, we performed multivariate time-dependent Receiver Operating Characteristic (ROC) analysis adjusted for confounding factors and compared the area under the curve (AUC) values. Additionally, we performed subgroup analyses to examine the effects of nutritional indicators, frailty, surgical procedures, and pre- and postoperative adjuvant chemotherapy. Finally, a nomogram was developed to establish a predictive model for survival probability based on 6MWD-CCI. Statistical significance was set at p < 0.05. All analyses were performed using EZR on R commander ver. 1.55 (Saitama Medical Center, Jichi Medical University, Japan) ^(18)^.

## Results

### Characteristics of participants

The baseline characteristics of the participants are shown in Table 1. The median age was 72 years, with a slightly higher proportion of females. The median 6MWD was 430 m (interquartile range: 390-470 m), with 25 participants (29.4%) failing to reach 400 m. Regarding the CCI, 22 participants were classified as low, 54 as middle, and nine as high. No significant correlation was observed between 6MWD and CCI (correlation coefficient −0.037, p = 0.739). Furthermore, 6MWD distribution showed no significant differences across the three CCI categories (p = 0.274). Clinical stage II was the most prevalent, accounting for 80 participants (94.1%). Postoperative complications occurred in 20.8% of cases, affecting approximately one in five participants.

**Table 1.** Baseline Characteristics of the Patients (N = 85).

Variable	Value
Age (years)	72 (70.0-76.0)
Male/female, n (%)	39 (45.9) / 46 (54.1)
BMI (kg/m^2^)	22.4 (20.5-24.3)
GNRI	95.4 (89.5-101.8)
Brinkman Index	15.0 (0-765)
NAC, n (%)	73 (85.9)
CCI, n (%)	
Low	22 (25.9)
Middle	54 (63.5)
High	9 (10.6)
Clinical stage, n (%)	
I	5 (5.9)
II	80 (94.1)
Grip strength (%)	40 (34.6-41.4)
KES (N/kg)	3.25 (2.74-3.33)
6MWD (m)	430 (390.0-470.0)
Clinical Frailty Scale	3 (2-3.25)
Pulmonary function
FEV1% (%)	76.6 (69.9-81.1)
%VC (%)	107.4 (93.4-117.9)
Hb (g/dL)	11.4 (10.6-11.9)
TP (g/dL)	6.3 (6.0-6.6)
Alb (g/dL)	3.6 (3.2-3.8)
Surgical procedure	
PD/DP, n (%)	50 (58.8) / 35 (41.2)
Operative time (min)	321.0 (253.0-399.0)
Infusion volume (ml)	3010.0 (2110.0-4255.0)
Blood loss (ml)	320.0 (150.0-565.0.3)
Complications, n (%)	15 (20.8)
6MWD-CCI Low-risk	2 (13.3)
Middle-risk	9 (60.0)
High-risk	4 (26.7)
LOS（days）	21.0 (17.0-28.0)

Values are reported as the median (interquartile range) or number of patients (percentage). %VC: percent vital capacity; 6MWD-CCI: the combination of 6MWD and CCI; Alb: serum albumin; BMI: body mass index; CCI: Charlson Comorbidity Index; DP: distal pancreatectomy; FEV1%: percent forced expiratory volume in 1 s; GNRI: geriatric nutritional risk index; Hb: hemoglobin; KES: knee extension strength; LDP: laparoscopic distal pancreatectomy; LOS: length of hospital stay; NAC: neoadjuvant chemotherapy; PD: pancreatoduodenectomy; TP: total pancreatectomy.

### Overall survival

**Figure 1.**
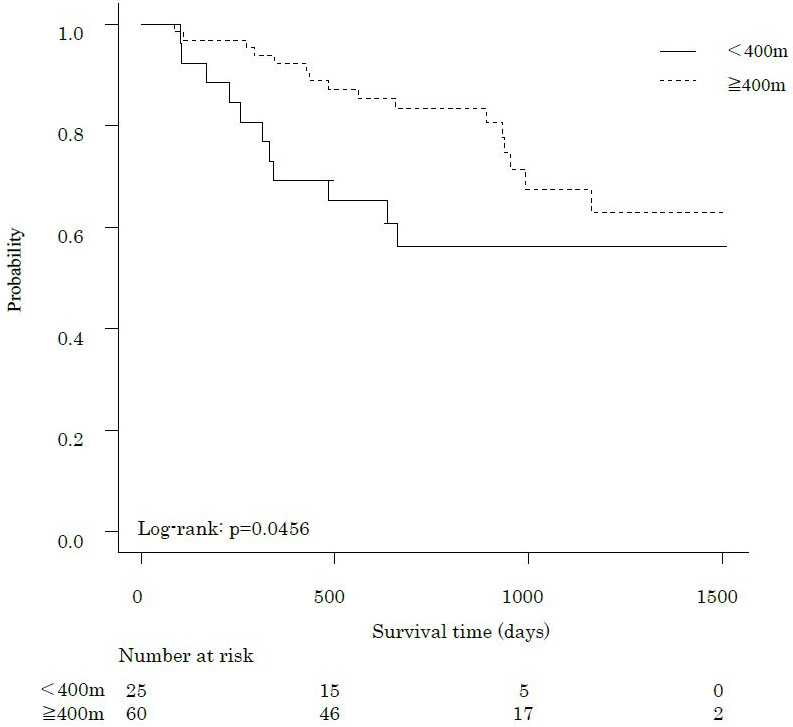
Kaplan-Meier survival curves for the overall survival after pancreatectomy in patients with pancreatic cancer, according to the preoperative 6-min walk distance. Kaplan-Meier survival curve showing survival probability based on a 6MWD cut-off of 400 m. The dashed line represents patients with a 6MWD of ≥400 m, whereas the solid line represents those with a 6MWD <400 m. 6MWD, 6-minute walk distance.

**Figure 2.**
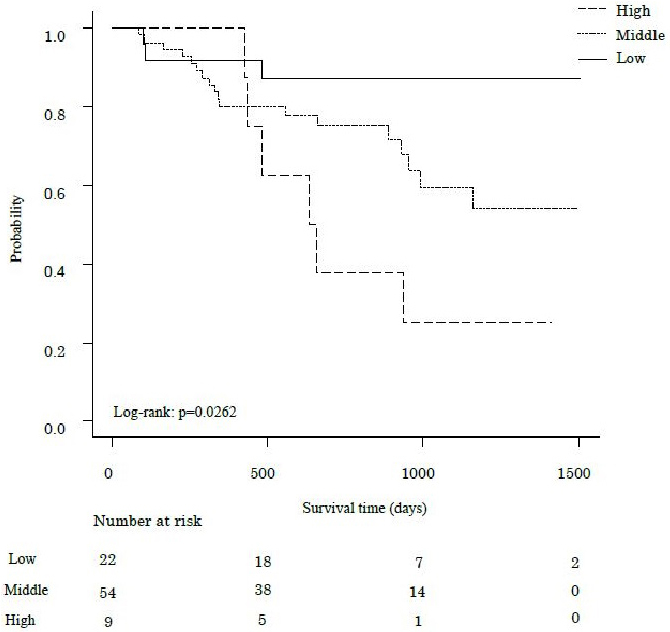
Kaplan-Meier analysis of survival rates in patients with pancreatic cancer, stratified by risk categories based on the CCI. Survival rates of patients with pancreatic cancer are categorized by the CCI: low, middle, and high. Patients with a high CCI had significantly lower survival rates compared to those in the other groups (p < 0.05). CCI, Charlson Comorbidity Index.

**Figure 3.**
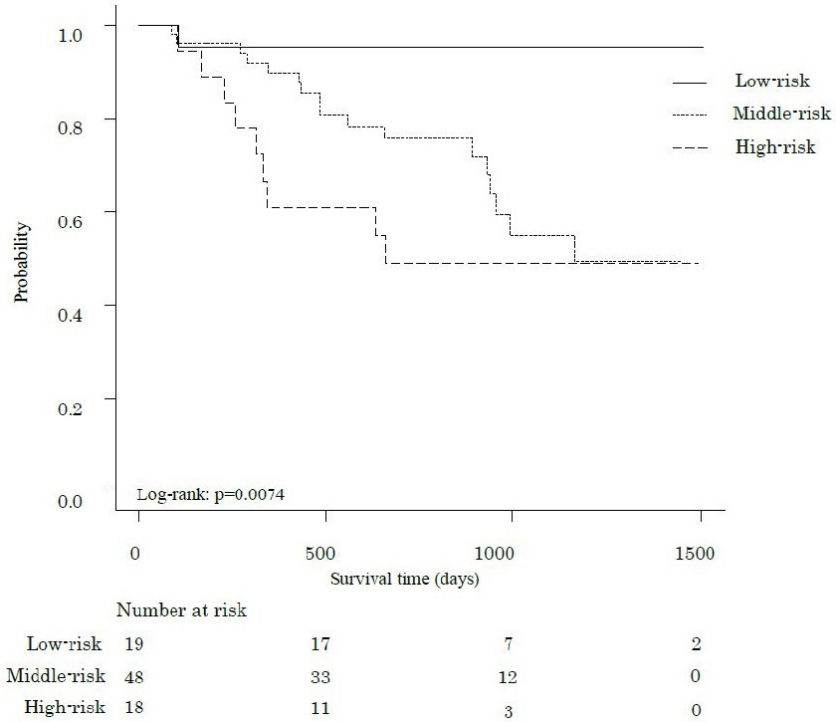
Kaplan-Meier survival curves for the overall survival after pancreatectomy in patients with pancreatic cancer, according to the preoperative 6MWD-CCI. Survival rates of patients with pancreatic cancer stratified by a combination of 6MWD and the CCI. Patients in the high group demonstrated significantly lower survival rates than those in other groups (p < 0.05). 6MWD, 6-minute walk distance; CCI, Charlson Comorbidity Index.

The median follow-up period was 802 days, during which 27 participants (31.8%) died. Figure 1, 2 and 3 show the comparative survival rates for each parameter. Participants with a 6MWD of less than 400 m had significantly lower survival rates than those with a 6MWD of 400 m or more (p < 0.05). Survival rates also decreased with increasing CCI scores, with the high group showing the poorest outcomes, followed by the middle and low groups (p < 0.05). Similarly, for the 6MWD-CCI, survival rates decreased in the order of high-risk, middle-risk, and low-risk groups (p < 0.05).

### Association between various indicators and survival rates

**Figure 4.**
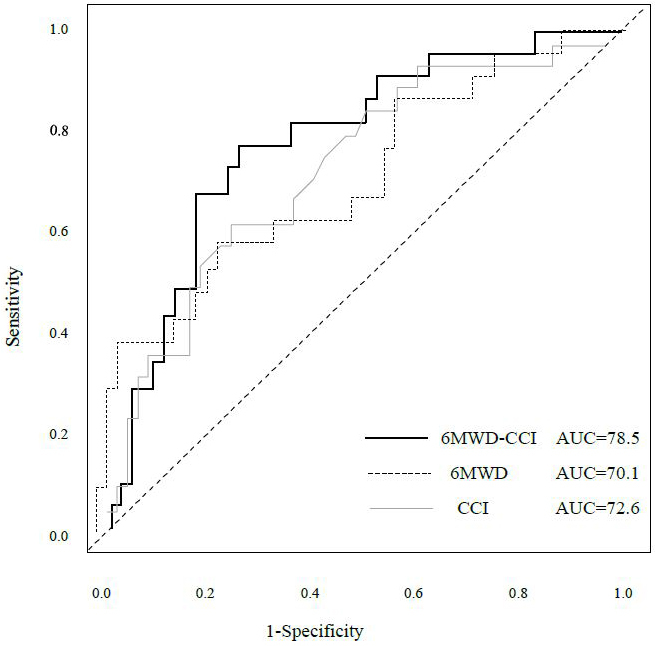
Predictive performance comparison by time-dependent AUC: 6MWD-CCI composite versus individual components (multivariable-adjusted). Multivariable ROC curves show the predictive performance of 6MWD-CCI, 6MWD, and CCI after adjustment for age, sex, and clinical stage. The composite 6MWD-CCI score demonstrated the highest discriminative ability (AUC=0.785, 95% CI XX-XX), followed by CCI (AUC=0.726) and 6MWD alone (AUC=0.701). 6MWD, 6-minute walk distance; AUC, area under the curve; CI, confidence interval; CCI, Charlson Comorbidity Index.

**Figure 5.**
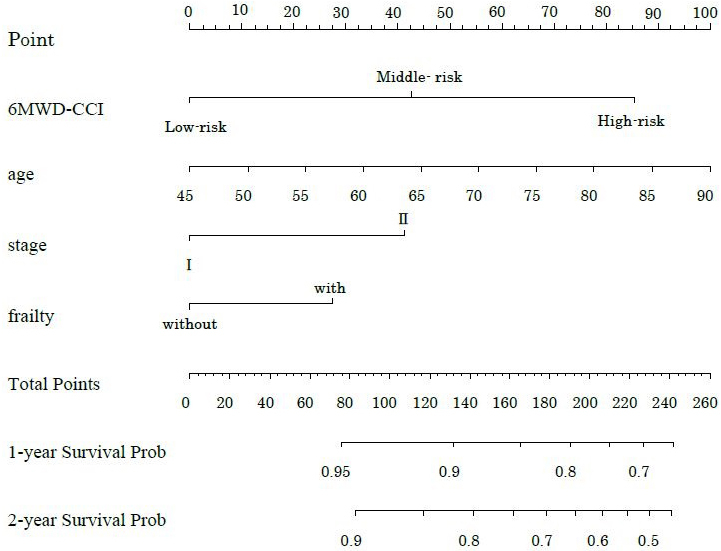
proposed Nomogram for predicting 1-year and 2-year survival probability of patients with pancreatic cancer. 6MWD-CCI, the combination of 6MWD and CCI. Nomogram incorporating 6MWD, CCI, and other patient characteristics to calculate individual and total scores. The graph highlights the significant impact of 6MWD-CCI on survival rates, as shown by its contribution to the total score. 6MWD, 6-minute walk distance; CCI, Charlson Comorbidity Index.

Table 2 shows the hazard ratios (HRs) for overall survival and 95% confidence intervals (CIs) derived from the Cox proportional hazards model. Model 1 included age, clinical stage, surgical procedure, and postoperative complications as adjustment variables. A 6MWD below 400 was significantly associated with survival (HR 2.80, 95% CI 1.26-6.22, p = 0.01). Among the CCI categories, only the high group demonstrated significance (HR 7.25, 95% CI 1.69-31.0, p = 0.07). For 6MWD-CCI, both the middle-risk and high-risk groups showed significant associations with survival (middle-risk: HR 9.83, 95% CI 1.27-76.32, p = 0.029; high-risk: HR 15.20, 95% CI 1.88-108.6, p = 0.011). Model 2, which included age, clinical stage, surgical procedure, and frailty as adjusted variables, showed no significant association for 6MWD (HR 2.45, 95% CI 0.94-6.37, p = 0.066). Only the high CCI category showed significance (HR 4.55, 95% CI 1.04-19.97, p = 0.045). The 6MWD-CCI showed similar results to those observed in Model 1 (middle-risk: HR 6.79, 95% CI 1.04-59.46, p = 0.043; high-risk: HR 10.07, 95% CI 1.18-85.74, p = 0.035). Figure 4 presents the time-dependent multivariate ROC curves. The AUC values were 0.785 for 6MWD-CCI, 0.701 for 6MWD, and 0.726 for CCI. Furthermore, in addition to 6MWD-CCI, we examined the association between survival rates and both GNRI and frailty as outcome variables, but only 6MWD-CCI showed a significant correlation (Supplementary Table 1). A sub-analysis comparing pancreatoduodenectomy and distal pancreatectomy is presented in Supplementary Table 2 and Supplementary Figure 1. Minimally invasive surgery was performed in only one case, with most cases involving open surgery. The operative time differed significantly between the groups; however, the survival rates showed no significant differences. While we presented the implementation and adherence rates for both neoadjuvant and adjuvant chemotherapy, our study found no significant impact on survival rates (Supplementary Table 3). Figure 5 shows the nomogram-predicted probabilities for patients with pancreatic cancer, calculating individual scores to determine the total score.

**Table 2.** Association of Each Indicator and Overall Survival in Patients with Pancreatic Cancer According to Cox Regression Models Adjusted for Potential Confounders.

		Model 1		Model 2	
	n	HR (95% CI)	*p*-value	HR (95% CI)	*p*-value
6MWD					
≧400	60	Ref.		Ref.	
< 400	25	2.80 (1.26-6.22)	0.01	2.45 (0.94-6.37)	0.066
CCI					
Low	22	Ref.		Ref.	
Middle	54	3.21 (0.91-11.27)	0.07	2.37 (0.70-8.09)	0.167
High	9	7.25 (1.69-31.0)	< 0.01	4.55 (1.04-19.97)	0.045
6MWD-CCI					
Low-risk	19	Ref.		Ref.	
Middle-risk	48	9.83 (1.27-76.32)	0.029	6.79 (1.04-59.46)	0.043
High-risk	18	15.20 (1.88-108.6)	0.011	10.07 (1.18-85.74)	0.035

Data are presented as hazard ratios (95% confidence intervals). Model 1: Adjusted for age, clinical stage, surgical procedure and postoperative complications. Model 2: Adjusted for age, clinical stage, surgical procedure and frailty. 6MWD: 6-min walk distance; 6MWD-CCI: the combination of 6MWD and CCI.CI: confidence interval; CCI: Charlson comorbidity index.

## Discussion

This study examined the relationship between survival in patients with pancreatic cancer and three key factors: preoperative exercise tolerance, comorbidities, and their combined effect. Patients classified as high-risk based on 6MWD, CCI, and 6MWD-CCI exhibited significantly lower survival rates compared to low-risk groups. Although individual parameters did not demonstrate significant associations with survival after adjusting for confounders in multivariable analysis, the 6MWD-CCI remained independently associated with survival, indicating its robustness as a prognostic factor.

As the CCI score increases, mortality rates also rise, with additional metrics enhancing the accuracy of mortality predictions ^(12)^. Our findings align with prior research. Many patients present with pre-existing comorbidities before surgery, underscoring the importance of the CCI in risk stratification. Organ dysfunction influences treatment planning and outcomes ^(19)^, with the prevalence of comorbidities increasing irreversibly with age ^(20)^. The age-adjusted CCI (ACCI), developed in 1994 by including age as a factor ^(21)^, has become a widely used prognostic tool in pancreatic cancer ^(22), (23)^. However, some studies have suggested that clinical staging and PS are superior predictors compared to ACCI ^(24)^. The high weighting of age in the ACCI may overestimate risk, particularly in aging societies like Japan. Therefore, differentiating between the use of CCI and ACCI may be necessary depending on the study design and the characteristics of the target population. The 6MWD-CCI composite offers distinct clinical advantages as a simple and rapidly assessable tool that can be immediately adopted in routine practice. Integration with electronic health record systems or clinical pathways could enable automated score calculation, facilitating risk stratification and supporting treatment decision-making. This metric may be particularly valuable for patients with multiple comorbidities, where single-parameter assessments often prove inadequate - the multidimensional evaluation provided by 6MWD-CCI addresses this critical gap. Cardiopulmonary exercise testing is a standard method for evaluating exercise tolerance, with the ventilation/carbon dioxide output ratio slope recognized as a long-term outcome predictor ^(25)^. However, due to its high cost and equipment requirements, the 6MWT offers a practical alternative in clinical settings. The 6MWT assesses oxygen utilization and transport mechanisms, including pulmonary, cardiac, and vascular functions ^(26), (27)^. A longer 6MWD moderately correlates with higher peak oxygen uptake and improved physical performance ^(28)^. Patients with pancreatic cancer often exhibit reduced cardiopulmonary function and muscle strength even at early treatment stages ^(29)^, with low preoperative exercise tolerance contributing to poorer outcomes ^(28)^. Conversely, maintaining high physical reserves preoperatively may help preserve cardiopulmonary function and PS, enabling tolerance to postoperative chemotherapy and other treatments. Unlike the CCI, the 6MWD is a modifiable factor, making it critical for assessing and enhancing preoperative functional reserves.

In this study, age, clinical stage, surgical procedure, postoperative complications, and frailty were included as confounding factors. The 5-year survival rates for patients with pancreatic cancer by disease stage are reported as 45.8% for stage I, 20.4% for stage II, 6.1% for stage III, and 1.3% for stage IV, with a sharp decline from stage III ^(1)^. Restricting the analysis to stages I and II likely minimized bias. Furthermore, postoperative complications have been associated with 6MWD in previous studies ^(6)^, and this risk is known to be influenced by comorbidities ^(22)^. Frail patients often have multiple comorbidities ^(30)^, which are inherently linked to exercise tolerance. Previous research has identified preoperative frailty as a predictor of postoperative complications ^(31)^. By constructing separate models accounting for frailty and postoperative complications while managing multicollinearity, reliable results were achieved. Patients with frailty and low physical reserves struggle to compensate for postoperative tissue hypoxia and dysfunction, leading to prolonged surgical stress and complications ^(32)^. Prolonged hospital stays and unplanned readmissions associated with complications adversely affect long-term outcomes ^(33)^. Therefore, preventing frailty from the preoperative stage is essential. Finally, age was included in the analysis as a standard confounding factor. These variables represent important determinants of 6MWD and CCI from distinct perspectives: baseline characteristics, disease profiles, treatment effects, and physical status. Due to sample size limitations, we restricted the number of adjustment variables to ensure robust results. Future studies with larger cohorts should incorporate more detailed confounding factors. The lack of observed association between chemotherapy adherence and survival may be attributed to several factors. The high implementation and completion rates of NAC (85% and 84%, respectively) and adjuvant chemotherapy (91.8% receipt rate) likely limited our ability to detect survival differences due to reduced variability between groups. Even among patients who discontinued adjuvant therapy (39.7%), the initiation of treatment itself may have provided some survival benefit. Additionally, the predominance of Stage I-II patients in our cohort, who typically have better prognoses, may have made chemotherapy effects harder to distinguish. Future studies should explore stage-specific effects, treatment duration impacts, and factors influencing adherence, such as side effect management, as these could provide more clinically actionable insights. Previous studies have explored survival rates using parameter combinations similar to those in this study ^(34), (35)^. These investigations highlight the association between long-term outcomes and combinations of physical factors, such as handgrip strength, calf circumference, and nutritional status. Nutritional status, like 6MWD ^(36)^, is a modifiable factor and a significant predictor. Future research integrating nutritional status into prognostic models is crucial. This study had several limitations. Firstly, this was a single-center, retrospective study with a limited sample size, which may restrict the statistical power and generalizability of our findings. Secondly, critical factors influencing survival, such as details of unplanned readmissions, were not examined. Future multicenter studies with larger cohorts are needed to validate these findings, including long-term follow-up with 5-year survival data. In conclusion, the combination of preoperative exercise tolerance and comorbidities in pancreatic cancer was a more robust predictor of survival than either parameter alone. The study highlights the importance of integrating both physical and systemic health factors to enhance risk stratification. Implementing the 6MWD-CCI into clinical practice may contribute to improved surgical outcomes and long-term prognosis in patients with pancreatic cancer.

## Article Information

### Conflicts of Interest

None

### Acknowledgement

We would like to thank Editage (www.editage.jp) for English language editing.

### Author Contributions

Data collection: Makoto Onji and Asuka Nakai. Data analysis & organization and manuscript writing: Makoto Onji. Accountability and Manuscript revision: Shingo Kozono, Shinji Kakizoe, and Koichi Naito. All authors read and approved the final manuscript.

### Approval by Institutional Review Board (IRB)

Approval No. 202401006. Kitakyushu Municipal Medical Center.

## Supplement

Supplementary Materials Supplementary Figure 1. Comparison of survival rates between PD and DP using Kaplan-Meier curves and the log-rank test.
