# Supplementary material for: Association between Preoperative Exercise Tolerance, Comorbidities, and Survival Rates in Patients with Pancreatic Cancer
Source: JMA J. 2025 Jun 20;8(3):893–902. doi: 10.31662/jmaj.2025-0105 (PMC12329037; doi:10.31662/jmaj.2025-0105)
Supplement: Supplementary Materials — Supplementary Figure 1. Comparison of survival rates between PD and DP using Kaplan-Meier curves and the log-rank test. [file 2433-3298-8-3-0893-s001.pdf]

Supplementary Table 1.

Comparison of 6MWD-CCI with conventional prognostic indicators using cox proportional hazards analysis

	HR	95% CI	*p*-value
Model 1.			
6 MWD-CCI	2.38	1.28 – 4.42	< 0.01
GNRI	1.55	0.70 – 3.41	0.28
Model 2.			
6 MWD-CCI	2.27	1.15 – 4.51	< 0.05
Frailty	1.24	0.52 – 2.92	0.62

Adjusted for age, sex and clinical stage

6MWD-CCI, the combination of 6MWD and CCI, GNRI: geriatric nutritional risk index

Supplementary Table 2. Comparison of factors between groups PD and DP

Variable		PD (n=50)	DP (n=35)	*p*-value
Age (y)		73.0 (70.0–77.8)	72.8 (69.5–82.0)	0.285
Male/female, n (%)		21(42.0) / 29 (58.0)	18 (51.4) / 17 (48.6)	0.507
GNRI		94.1 (85.1–101.1)	97.5 (93.3–104.6)	0.019
NAC, n (%)		43 (86.0)	30 (85.7)	1
CCI, n (%)				0.638
	Low	11 (22.0)	11 (31.4)	
	Middle	33 (66.0)	21 (60.0)	
	High	6 (12.0)	3 (8.6)	
Cancer stage, n (%)				1
	I	3	2	
	II	47	33	
KES (N/kg)		3.25 (3.0–6.7)	3.25 (2.56–3.26)	0.341
6 MWD (m)		430.0 (390.0–450.0)	435.0 (387.5–483.5)	0.506
Clinical frailty scale		3 (1–4)	3 (2–3)	0.417
Approach, OS/MIS, n (%)		50 (100) / 0 (0)	34 (97.1) / 1 (2.9)	0.857
Operative time (min)		371.0 (320.3–464.8)	248.0 (184.0–294.0)	< 0.01
Complications, n (%)		12 (24.0)	12 (34.3)	0.335

LOS (days)	21 (17.3-28.0)	21.0 (16.0-29.0)	0.851
Death, n (%)	20 (40)	7 (20)	0.061

Values are reported as the median (interquartile range) or number of patients (percentage). PD: pancreatoduodenectomy, DP: distal pancreatectomy, GNRI: geriatric nutritional risk index, NAC: neoadjuvant chemotherapy, CCI: Charlson Comorbidity Index, KES: knee extension strength, 6 MWD: 6-minute walk distance, OS: open surgery, MIS: minimally invasive surgery, LOS: length of hospital stay.

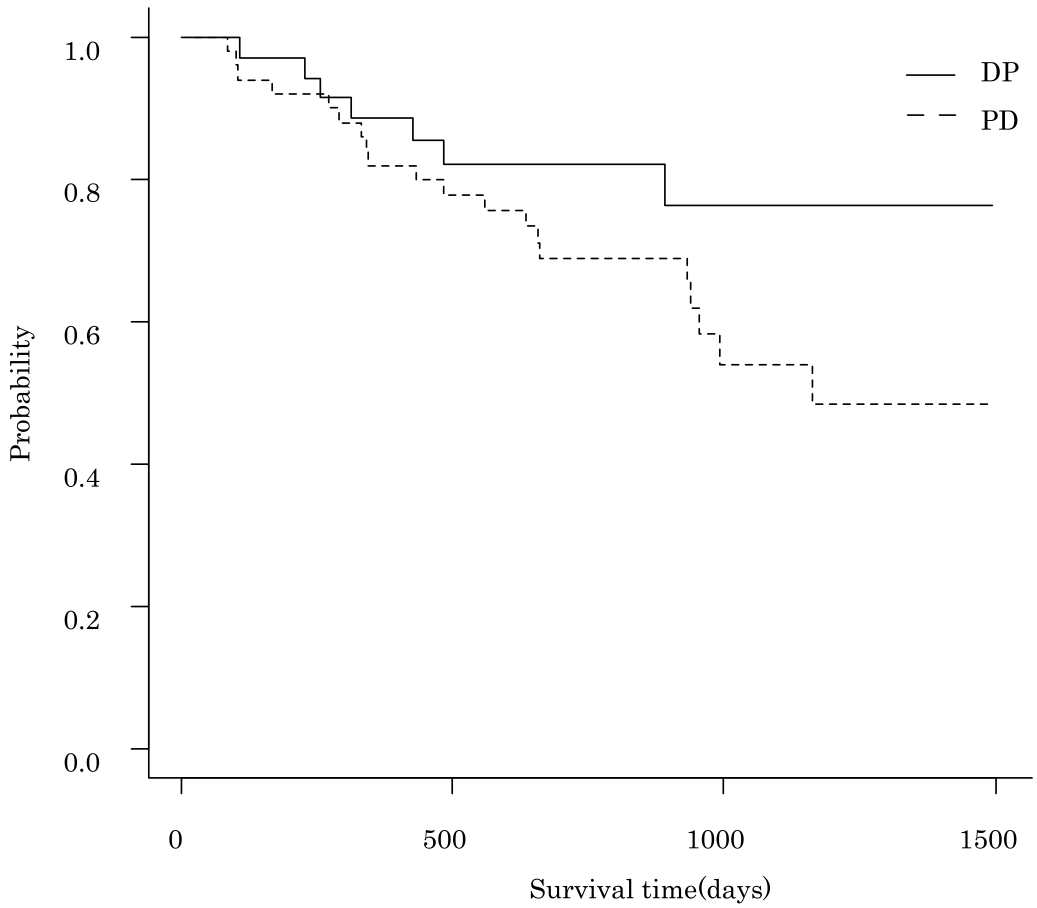

Number at risk

DP	35	24	10	0
PD	50	37	12	2

Supplementary Figure.1

Supplementary Table 3

Association Between Treatment Adherence and Survival Outcomes in Patients Receiving Neoadjuvant and Adjuvant Chemotherapy

Category	n (%)	Survived (n)	Deceased (n)	*p*-value
NAC	73 (85.0)	51	22	
Completed	63 (84.0)	44 (86.3)	19 (86.4)	1
Discontinued	10 (16.0)	7 (13.7)	3 (13.6)	
Dose adjustment during NAC		51	22	
No dose reduction	49 (67.1)	34 (66.7)	15 (68.2)	1
Dose reduction	24 (32.9)	17 (33.3)	7 (31.8)	
Received adjuvant chemotherapy	78 (91.8)	58	27	
Completed	47 (60.3)	35 (60.3)	12 (44.4)	0.295
Discontinued	31 (39.7)	20 (34.5)	14 (51.9)	
Not received	7 (8.2)	3 (5.1)	1 (3.7)	

NAC: neoadjuvant chemotherapy
